# Bispecific Antibodies: Strategies Available to Optimize Their Safe Delivery in Patients with Multiple Myeloma

**DOI:** 10.3390/antib15010005

**Published:** 2026-01-05

**Authors:** Hannah Victoria Giles, Bhuvan Kishore

**Affiliations:** 1 Department of Clinical Haematology, University Hospitals Birmingham NHS Foundation Trust, Birmingham B15 2TH, UK; bhuvan.kishore@nhs.net; 2 Birmingham Health Partners Fellow, College of Medicine and Health, University of Birmingham, Birmingham B15 2TT, UK

**Keywords:** bispecific antibodies, multiple myeloma, immunoglobulin prophylaxis, CRS, tocilizumab prophylaxis

## Abstract

Bispecific antibodies (BsAbs) have emerged as an important new class drugs for the treatment of multiple myeloma (MM) over the last few years. Currently, BsAbs are only licensed for use as monotherapy in patients with relapsed/refractory MM who have had at least three prior lines of treatment and are triple class-exposed (patients who have received an anti-CD38 monoclonal antibody, an immunodulatory drug, and a proteasome inhibitor). However, their use in earlier lines, including in the upfront setting, is being explored in multiple ongoing clinical trials with promising early results. The BsAbs have specific toxicities, including a high rate of low-grade cytokine release syndrome and, less commonly, immune effector cell-associated neurotoxicity syndrome. These immune-related toxicities occur almost exclusively during the initiation phase of the BsAbs. This has led to frequent hospitalization of patients for the duration of the initial step-up dosing phase. Strategies that could facilitate outpatient step-up dosing, such as tocilizumab prophylaxis, will become even more critical if BsAbs move into earlier lines of treatment and are used in larger numbers of patients. Optimizing infection prophylaxis is critical for ensuring the safe delivery of BsAbs as infection is the leading cause of non-relapse mortality in patients being treated with BsAbs. Multiple strategies to minimize the infection risk, including antimicrobial prophylaxis, immunoglobulin replacement, vaccination and reduced dosing frequency, have been evaluated. The clinical data on the efficacy of these supportive measures are described in this review article alongside the available strategies for mitigating and managing CRS and ICANS.

## 1. Introduction

Multiple myeloma (MM) is a neoplastic plasma cell disorder which, despite therapeutic advances, remains incurable. Bispecific antibodies (BsAbs) have emerged as promising agents with overall response rates (ORR) of 61–74% in patients with relapsed/refractory MM (RRMM) who have previously been treated with proteasome inhibitors, immunomodulatory drugs, and anti-CD38 antibodies [1,2,3,4]. These patients are referred to as being triple-class exposed (TCE). The antigenic targets and efficacy data of the BsAbs licensed for use in patients with RRMM are summarized in Table 1.

BsAbs simultaneously bind a tumor antigen on the MM cells, such as BCMA or GPRC5D, and CD3 on T-cells. Additional antigenic targets, such as FcRH5, are being explored as well as dual targeting antibodies that bind two tumor antigens and CD3 simultaneously [6]. The simultaneous binding of an antigen on the MM cells and CD3 on the T-cells enables the formation of an “immunological synapse” and leads to T-cell activation, proliferation, and degranulation. The activated T-cells release perforin, which creates pores in the MM cell membranes. Granzyme B enters the MM cells through these pores and induces apoptosis of MM cells via caspase activation [7].

Several BsAbs—teclistamab, elranatamab, linvoseltamab, and talquetamab—have received regulatory approval for the treatment of TCE patients with RRMM. The off-the-shelf availability of BsAbs contrasts with CAR T-cell therapies, which are resource-intensive and time-consuming. This means that the BsAbs can be administered quickly and to a broader population. Owing to their relative convenience and the increasing frequency of patients becoming TCE within 1–2 lines of treatment, BsAbs are being explored in earlier lines of treatment and in combination with other anti-MM drugs with encouraging results [8,9,10,11,12,13,14,15,16].

Like CAR T-cell products, BsAbs can induce immune-related toxicities including cytokine release syndrome (CRS) and immune effector cell-associated neurotoxicity syndrome (ICANS), although severe cases are much less common. The risk of CRS and ICANS have important implications for the delivery and monitoring of patients being treated with these agents. As a class, the BsAbs also carry a significant risk of infection and infection is the leading cause of non-relapse mortality in RRMM patients being treated with BsAbs. This article reviews the nature and incidence of these complications and suggests practical solutions to mitigate and address these adverse effects.

## 2. CRS

CRS is a systemic hyperinflammatory response that is characterized by fever, hypotension, and hypoxia and can lead to organ dysfunction [17]. CRS occurs due to the activation and proliferation of multiple immune cell types, including T-cells, B cells, NK cells, macrophages, and dendritic cells and the subsequent release of pro-inflammatory cytokines. IL-6 signaling plays a central role in the pathophysiology of CRS by activating vascular endothelial cells. Endothelial cell activation triggers the secretion of further pro-inflammatory cytokines and drives a self-perpetuating feedback loop. This cascade of events leads to increased vascular permeability, which can lead to hypotension, pulmonary edema, and acute respiratory distress syndrome [18,19].

### 2.1. Grading and Management of CRS

The severity of CRS is graded according to international consensus criteria based on the patient’s temperature, oxygen saturations, and blood pressure (Table 2). The management of CRS is guided by the severity and duration of symptoms and ranges from supportive measures with antipyretics to anti-IL6 directed therapy with tocilizumab and corticosteroids, and severe cases may require anti-TNF therapy and organ support in the intensive care unit (Table 3) [17,20].

To reduce the risk and severity of CRS, BsAbs are initiated with a step-up dosing phase. Step-up dosing typically involves administering increasing doses over the first two to three doses prior to delivery of the first full-dose of the BsAb. This approach is designed to initiate T-cell engagement and cytokine release in a controlled manner when disease burden is at its highest. Pre-medication with corticosteroids, antipyretics, and antihistamines are also used during the step-up dosing phase to reduce the risk of CRS. Despite these measures, CRS is still commonly seen in patients treated with BsAbs, although most cases are low-grade. The rates of CRS reported in the registration trials for the BsAbs being used in routine clinical practice are summarized in Table 4. The incidence of CRS in patients treated with BsAbs in real-world series is comparable to the rates reported in the registration trials [1,2,5,21,22,23].

CRS typically occurs within hours to a small number of days of the step-up doses or first-full dose and is rare beyond this time frame [22]. Owing to this, risk patients need to be monitored at least once daily for CRS during step-up dosing. Guidance in terms of the specific time frame for each product is provided in their summary of product characteristics.

### 2.2. Outpatient Delivery of Step-Up Dosing

**Figure 1:**
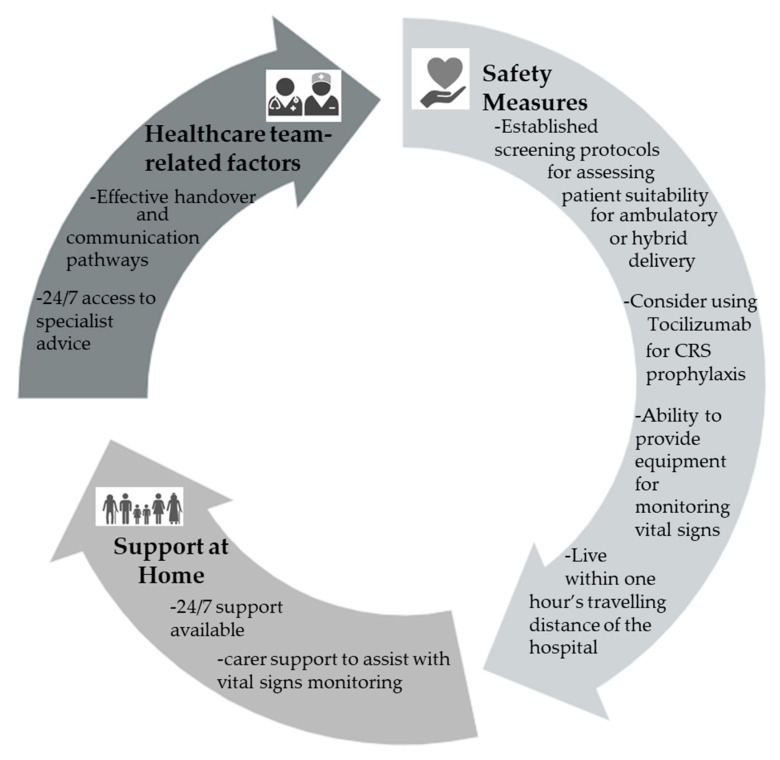
Factors to consider when evaluating patient suitability for outpatient delivery of BsAb step-up dosing.

Although the requirement for daily assessments and the fast administration time of the subcutaneous preparations make these agents suitable for initiation in the outpatient setting, many institutions are hospitalizing patients for the entire duration of the BsAb step-up dosing. The factors that should be considered when assessing patient suitability for outpatient delivery of BsAbs are summarized in Figure 1.

The low uptake of outpatient delivery of the step-up dosing phase is driven by the high incidence of CRS and variations in direct access to haemato-oncology services where specialist assessment and treatment of CRS can be initiated in a timely manner. This is a particular issue for centers where the haemato-oncology day unit is only open during weekdays.

In a survey of 19 centers across the United States of America, only 5/19 were delivering the step-dosing of teclistamab as a fully outpatient or hybrid program. In addition, 4/5 of the centers that were delivering the step-up dosing in an outpatient or hybrid setting hospitalized all patients with any grade of CRS. The single center managing patients with grade 1 CRS in an outpatient setting was the only center that had the ability to deliver tocilizumab in the outpatient setting [24].

In an effort to overcome the barrier of concerns over outpatient management of CRS, the use of prophylactic tocilizumab has been explored and has been shown to reduce the overall incidence of CRS to 0–26.3%, although it does not reduce the incidence of grade 2 CRS [23,25,26,27,28,29,30]. Importantly, the use of prophylactic tocilizumab does not have any adverse effect on the efficacy of the BsAbs in laboratory or clinical studies [25,29,30,31].

Despite the encouraging results with the use of prophylactic tocilizumab to facilitate the delivery of step-up performance in an outpatient or hybrid model, implementation has been limited to date [24]. The infrequent utilization of this prophylactic strategy is largely driven by the financial cost and reimbursement concerns as this is an off-license use of tocilizumab. However, Puttkammer et al. reported that exclusive outpatient delivery of teclistamab and talquetamab can provide cost savings, with a medication margin of USD 115,004 [23]. The ability to manage grade 1 CRS without tocilizumab is another important factor, with many centers managing this with either antipyretics alone or dexamethasone.

The benefits of an ambulatory or hybrid model for the delivery of the step-up dosing for BsAbs go beyond financial gains. These non-cost-saving benefits include reducing the risk of hospital-acquired infections; patients being able to spend more time in their own home with friends and family; and less inpatient bed utilization. Whilst the BsAbs are currently only being used in later lines, the patient numbers are smaller as there is significant attrition across the lines of therapy, with only around 60% of patients receiving treatment beyond third-line [32]. However, with patients becoming TCE earlier in their treatment journeys and the possibility that BsAbs may be more efficacious in earlier lines when T-cell fitness will be greater, larger numbers of patients may be treated with BsAbs in earlier lines in the future [8,9,10,11,12,13,14,15,33]. This will lead to significant bed pressures in already stretched healthcare services if the majority of patients continue to be hospitalized for the duration of their step-up dosing.

Whilst predictive models for the risk of CRS have been developed for lymphoma patients being treated with BsAbs [34], research is ongoing regarding which biomarkers and patient characteristics could be used to predict CRS risk in MM patients being treated with BsAbs. Disease burden is a well-established risk factor for CRS in other settings, but baseline disease burden has not been reported to predict CRS incidence or severity in patients treated with elranatamab or teclistamab [35,36]. Ethnicity may be a risk factor as higher rates of CRS have been reported in MM patients from East Asia treated with BsAbs [37,38] but this requires further evaluation. A lower rate of CRS in patients who had previously been treated with a T-cell redirecting therapy was reported by Hamadeh et al., but this finding has not been consistent [39,40].

## 3. ICANS

ICANS is characterized by confusion, reduced consciousness level, aphasia, raised intracranial pressure, and seizures. It is thought to be caused by cytokine-mediated endothelial activation and disruption of the blood–brain barrier. It therefore typically occurs alongside CRS but can occur after CRS has resolved. ICANS can also rarely occur in the absence of CRS. The incidence of ICANS in patients treated with BsAbs is much lower than the incidence of CRS. Overall, ICANS occurs in 3.0–14% of patients treated with BsAbs and cases of ICANS grade 3 or above are rare [1,2,3,5,21,22,41,42].

### Grading and Management of ICANS

The severity of ICANS is graded using the ICE score (Table 5) and management is guided by the severity (Table 6). It is crucial that non-ICANS causes for any new onset neurological symptoms are evaluated concurrently. Toxic, metabolic, and infectious aetiologies should be excluded in all patients with new onset neurological symptoms. If an infectious etiology is being considered, a lumbar puncture should also be considered. A CT or MRI head should be performed in all patients with ICANS grade ≥ 2 to exclude cerebral edema or other acute abnormalities, such as a bleed, and an EEG should be performed.

## 4. Infection Risk in MM Patients Treated with BsAbs

Infection is the leading cause of non-relapse mortality in patients treated with BsAbs [21,43]. Across clinical trials and real-world data series, infections of any grade have been reported in 42–80.0% of patients and grade 3 or 4 infections have been reported in 22–56% of patients [1,2,22,41,43,44,45,46,47]. The highest rates of grade ≥ 3 infections are seen in patients treated with BsAbs in combination with other anti-MM agents and the lowest in patients treated with GPRC5D-targeting BsAbs. The rates of infection observed in the clinical trials of the BsAbs with regulatory approval are summarized in Table 7.

The principal drivers of infection are as follows: hypogammaglobulinaemia due to apoptosis of normal plasma cells due to on-target off-tumor effects of the BCMA-targeting BsAbs; T-cell exhaustion due to chronic T-cell engagement; the cumulative effect of multiple prior lines of immunosuppressive therapy; treatment and disease-related neutropenia; and frequent healthcare contacts that increase exposure to pathogens.

The upper and lower respiratory tract are the most common sites for infection and the most common pathogens are viruses and bacteria [2,22,41,43]. During any active infection, the BsAb should be paused and targeted treatment for the specific pathogen should be initiated promptly. To facilitate this, nasopharyngeal aspirates or respiratory secretions should be sent for PCR testing for respiratory viruses in all patients with symptoms consistent with a respiratory tract infection alongside cultures for microscopy, culture, and sensitivity. Patients with influenza should be treated with direct acting antivirals. The choice of agent between oseltamivir, zanamivir, and baloxavir is guided by local protocols. Follow-up testing should be considered in patients who have not responded after five days of oseltamavir treatment to exclude oseltamivir resistance. If there is concern for oseltamivir resistance, treatment with zanamvir or baloxavir should be considered [48,49,50,51]. All MM on BsAbs with COVID-19 infection should be regarded as high-risk for progression to severe disease. The treatments indicated for COVID-19 infection are guided by whether they have required hospitalization and the degree of hypoxia and are summarized in Table 8.

Viral reactivations, including cytomegalovirus and hepatitis B, have been reported in patients treated with BsAbs [2,43,46,54,55]. The clinical significance of many of the CMV reactivations is uncertain and has led to heterogeneity in terms of treatment in the absence of organ-related symptoms [20,54]. Symptomatic CMV infection should be treated first-line with valganciclovir or ganciclovir [20,51].

## 5. Strategies to Reduce the Risk of Infection in Patients Receiving BsAbs

### 5.1. Reducing BsAb Dosing Frequency or Total Treatment Duration

The risk of new grade ≥ 3 infections remains significant throughout treatment, reflecting the ongoing humoral and cellular immune impairment. Reducing the frequency of the BsAbs in responding patients can ameliorate this risk [46] and many of the ongoing trials are utilizing less frequent dosing schedules. Encouraging high rates of sustained remissions after stopping BsAbs have been reported and ongoing studies are also investigating fixed-duration BsAb treatment [56].

### 5.2. Immunoglobulin Replacement Therapy

Administration of intravenous or subcutaneous immunoglobulin replacement therapy reduces the risk of infection by addressing the treatment-induced humoral impairment [44,57,58,59,60]. Primary prophylaxis with immunoglobulin therapy has also been shown to improve overall survival in patients treated with BsAbs [44]. In patients treated with BCMA-targeting BsAbs, the polyclonal IgG typically falls to <1 g/L within the first 1–2 months of therapy and immunoglobulin replacement therapy should be initiated when the polyclonal IgG level has fallen below 4 g/L [20,46,61].

Whilst immunoglobulin supplementation has been shown to be important for optimizing the outcomes for patients receiving BsAbs, it is still an area with some controversy. Firstly, given the significantly lower infection risk in patients treated with talquetamab compared to the BCMA-targeting BsAbs, it is less clear whether immunoglobulin prophylaxis is required for patients receiving talquetamab with a polyclonal IgG level below 4 g/L. This is pertinent as although the polyclonal IgG level tends to fall in the first few months of treatment, the IgG levels improve to above baseline levels in the longer term in responding patients [4]. Secondly, it is not yet known how less frequent dosing of BsAbs impacts the need, dosing, and frequency of immunoglobulin supplementation. Thirdly, it is not known how long it takes for the polyclonal immunoglobulin background to recover after discontinuation of BsAb therapy and therefore how long immunoglobulin replacement needs to be continued after treatment when a BsAb is discontinued. Finally, there is heterogeneity in the type of immunoglobulin replacement being used. Whilst some centers have access to both subcutaneous and intravenous immunoglobulin replacement therapy, other centers only have access to intravenous immunoglobulin replacement. Intravenous immunoglobulin replacement involves long infusion times, which may have a significant impact on day unit capacity in the long term as well as increasing time toxicity (the time spent on the delivery of cancer treatment, monitoring of its efficacy, and the delivery treatments required to prevent or manage treatment-related adverse effects) for patients.

Further work is needed to address these areas of controversy and ensure that this important but limited and expensive resource is used most efficiently and appropriately. This will be crucial to ensure that supply issues do not prevent patients who need immunoglobulin replacement therapy from gaining access to it in a timely manner in the future.

### 5.3. Management of Neutropenia

Grade 3 or 4 neutropenia (neutrophils 0.5–1.0 × 10^9^/L and neutrophils < 0.5 × 10^9^/L, respectively) are seen in 21.0–65.5% patients [1,2,22,41,46]. Neutropenia can be managed supportively with G-CSF. A reduction in the dosing frequency should also be considered in responding patients with grade 4 neutropenia [20,62].

### 5.4. Antiviral Prophylaxis

All patients being treated with BsAbs should receive antiviral prophylaxis against herpes simplex virus and varicella zoster virus with either acyclovir or valaciclovir. Owing to the risk of hepatic failure with hepatitis B reactivation, hepatitis B core antibody testing should be performed in all patients prior to initiation of a BsAb. Any patients at risk of hepatitis B reactivation should receive entecavir prophylaxis throughout their BsAb treatment [20].

### 5.5. Vaccination

Live vaccinations are contraindicated in patients being treated with BsAbs, but non-live vaccinations are recommended and should ideally be given prior to treatment initiation. Antibody responses to vaccination are significantly impaired in patients being treated with BCMA-targeting BsAbs. In a study by Frerichs et al. of patients in a partial response or better on teclistamab, only 5.9% responded to *H. influenzae* vaccination, 0% of patients had detectable anti-SARS-CoV-2 spike antibodies, and only 7.1% responded to *S. pneumoniae* vaccination [59]. Although vaccines can also induce T-cell-mediated immunity, T-cell responses may also be impaired in the setting of BsAb treatment as even after monthly administration, most circulating T-cells remain bound to the BsAbs [63].

### 5.6. Antibacterial Prophylaxis

Antibacterial prophylaxis has been shown to reduce the risk of grade ≥ 3 infections [43]. However, this strategy is not universally employed. The TEAMM study of fluroquinolone prophylaxis for the first twelve weeks of treatment in patients with newly diagnosed MM showed a benefit in terms of the reduction in febrile episodes, but there is no randomized trial data of this strategy in patients treated with BsAbs. Fluroquinolones are associated with an up to seven-fold increased risk of tendon rupture and two-fold increased risk of heart valve problems [64,65]. This strategy should therefore be considered in line with local guidance and the risk benefit profile of this strategy should be evaluated on an individual patient basis.

### 5.7. Antifungal Prophylaxis

Although neutropenia is common with BsAbs, invasive fungal infections, excluding *Pneumocystis jirovecii*, are uncommon [43,46]. Routine antifungal prophylaxis is therefore not required in the absence of prolonged neutropenia [20]. All patients should be given prophylaxis against *Pneumocystis jirovecii*.

## 6. Conclusions

BsAbs represent an important therapeutic addition to the MM treatment armamentarium. CRS is common but usually low-grade, so it should not be seen as a complete contraindication to outpatient delivery. Further research is needed to identify the optimal model for delivery, which may differ according to the facilities available at different health centers and reimbursement models for supportive medications such as tocilizumab.

Infections are common, particularly in patients treated with BCMA-targeting BsAbs. Vigilant attention should therefore be paid to the optimal dosing frequency and supportive medications to minimize the risk of excessive non-relapse mortality due to infection.

## Figures and Tables

**Table 1:** Bispecific Antibodies Licensed for the Treatment of Patients with RRMM [1,2,3,4,5].

Bispecific Antibody	MM Cell Target Antigen	Antibody Structure	Clinical Trial	Licensed Indication	ORR	Median Progression-Free Survival	Median Overall Survival
Teclistamab	BCMA	Humanized IgG4-proline, alanine, alanine antibody	MajesTEC-1 (165 patients)	Monotherapy for patients with RRMM who are TCE and have received at least three prior lines of therapy by EMA and four prior lines of therapy by FDA and have demonstrated progressive disease (PD)	63.0%	11.3 months	22.2 months
Elranatamab	BCMA	IgG2 kappa antibody derived from two monoclonal antibodies	MagnetisMM-3 (123 patients)	Monotherapy for patients with RRMM who are TCE and have received at least three prior lines of therapy by EMA and four prior lines of therapy by FDA and have demonstrated PD	61.0%	17.2 months	24.6 months
Linvoseltamab	BCMA	Recombinant human IgG4-based BsAB	LINKER-MM1 (117 patients received the licensed dose of 200 mg)	Monotherapy for patients with RRMM who are TCE and have received at least three prior lines of therapy by EMA and four prior lines of therapy by FDA and have demonstrated PD	71.0%	Not reached after median follow-up of 14.3 months, 70.0% 12-month PFS	31.4 months
Talquetamab	GPRC5D	Humanized IgG4-proline, alanine, alanine antibody	MonumenTAL-1 (375 patients)	Monotherapy for patients with RRMM who are TCE and have received at least three prior lines of therapy by EMA and four prior lines of therapy by FDA and have demonstrated PD	74.0% for 0.4 mg/Kg dose 69.0% for 0.8 mg/Kg dose	7.5 months for 0.4 mg/Kg dose 11.2 months for 0.8 mg/Kg dose	Not reached. 76.0% 12-month OS for 0.4 mg/Kg 77.0% 12-month OS for 0.8 mg/Kg dose

**Table 2:** ASTCT Consensus Criteria for CRS [17].

Grade	Temperature	Hypotension	Hypoxia
1	≥38 °C	None	None
2	≥38 °C	Hypotension that responds to intravenous fluids	Hypoxia requiring low-flow oxygen via nasal cannula or blow-by
3	≥38 °C	Hypotension requiring a single vasopressor with or without vasopressin	Hypoxia requiring supplemental oxygen via high-flow nasal cannula, face-mask, non-rebreather mask or Venturi mask
4	≥38 °C	Hypotension requiring multiple vasopressors (excluding vasopressin)	Requiring positive pressure non-invasive ventilation or intubation and mechanical ventilation

**Table 3:** IMWG Recommendations for CRS Management [20].

ATSCT CRS Grade	Management
1	Supportive measures such as antipyretics Assess for infection Consider early Tocilizumab use Early Tocilizumab use is encouraged for patients with persistent grade 1 CRS (>24 h)
2	Grade 2–4 CRS should be managed as an inpatient Supportive care as needed with intravenous fluids and oxygen supplementation Tocilizumab 8 mg/Kg (maximum 3 doses in 24 h and maximum 4 doses in total) If no improvement following Tocilizumab, second-line treatment with dexamethasone should be considered
3	Supportive care as needed Tocilizumab 8 mg/kg (maximum 3 doses in 24 h and maximum 4 doses in total) and dexamethasone 10 mg every 6 h Transfer the patient to a high dependency unit or intensive care unit In refractory cases, consider high-dose steroids such as methylprednisolone and anakinra
4	Supportive care as needed Consider high-dose steroids such as methylprednisolone and anakinra The patients should be managed in the intensive care unit

**Table 4:** Incidence and Severity of CRS with the BsAbs Licensed for Use in Patients with RRMM [1,2,3,4].

BsAb	Trial Name and Patient Population	CRS, Any Grade	CRS Grade ≥ 3
Elranatamab	MagnetisMM-3 RRMM	57.7%	0%
Teclistamab	MajesTEC-1 RRMM	72.1%	0.6%
Linvoseltamab	LINKER-MM1 RRMM	46.2%	0.9%
Talquetamab	MONUMENTAL-1 RRMM	77.0% in 0.4 mg/Kg cohort 74.0% of 0.8 mg/Kg cohort	2.0% of 0.4 mg/Kg cohort 1.0% of 0.8 mg/Kg cohort

**Table 5:** ICE Scoring [17].

Domain	Questions	Points
Orientation	Year Month City Hospital	4
Naming	Name three objects	3
Following commands	Follow a simple command, e.g., show me three fingers	1
Writing	Write a simple sentence e.g., My favorite color is green	1
Attention	Count backwards from 100 to 0 in 10 s	1

**Table 6:** ATSCT Consensus Criteria for ICANS Grading [17].

Grade	ICE Score	Level of Consciousness	Seizures	Motor Findings	Raised ICP/Cerebral Edema
1	7–9	Awake spontaneously	None	Normal	None
2	3–6	Responds to voice	None	Normal	Normal
3	0–2	Responds to tactile stimuli	Rapidly resolving seizures or non-convulsive seizures on EEG that resolve with intervention	Normal	Focal edema on imaging
4	0	Only rousable with vigorous tactile stimuli or comatose	Seizures lasting > 5 min or repeated seizures without a return to baseline between seizure episodes	Deep focal motor weakness, e.g., hemiparesis	Diffuse cerebral edema; decerebrate or decorticate posturing; VIth cranial nerve palsy; or Cushing’s triad

**Table 7:** Infections Rates in Patients with RRMM Treated with BsAbs as Monotherapy [1,2,3,4].

BsAb	Trial Name	Any Grade Infections	Grade 3 or 4 Infections
Elranatamab	MagnetisMM-3	69.9%	39.8% (6.5% fatal)
Teclistamab	MajesTEC-1	76.4%	44.8%
Linvoseltamab	LINKER-MM1	74.4%	35.9%
Talquetamab	MONUMENTAL-1	59.0% in 0.4 mg/Kg cohort 68.0% of 0.8 mg/Kg cohort	20.0% of 0.4 mg/Kg cohort 18.0% of 0.8 mg/Kg cohort

**Table 8:** Management strategies for COVID-19 infection according to infection severity and care setting [52,53].

COVID-19 Infection Severity	Therapeutic Options
Mild to moderate COVID-19 infection with no hypoxia	Nirmatrelvir/ritonavir (Paxlovid) X 5 days (oral) Molnupiravir may be used second-line if Paxlovid is unsuitable Remdesivir for 3 days (intravenous) Sotrovimab can be considered after MDT discussion
Hospitalized patients with mild to moderate COVID-19 infection with no hypoxia	If at high risk for progression and within 7 days of symptom onset, Remdesivir for 3 days may be given if at high risk for progression and within 7 days of symptoms onset
Hospitalized for severe but not critical COVID-19 infection (hypoxia managed with low-flow supplemental oxygen)	Dexamethasone 6 mg/day for 10 days or until discharge Remdesivir for 5 days IL-6 inhibition with Tocilizumab or Sarilumab in progressive disease and CRP ≥ 75 mg/L or Baricitinib or tofacitinib in patients and CRP ≥ 75 mg/L
Hospitalized and Critically Unwell with COVID-19 Infection (hypoxia requiring high-flow nasal oxygen or non-invasive ventilation)	Dexamethasone 6 mg/day for 10 days or until discharge IL-6 inhibition with Tocilizumab or Sarilumab in progressive disease and CRP ≥ 75 mg/L Baricitinib or tofacitinib in patients and CRP ≥ 75 mg/L
Hospitalized for critically ill COVID-19, needing invasive mechanical ventilation or ECMO	Dexamethasone 6 mg/day for 10 days or until discharge IL-6 inhibition with Tocilizumab or sarilumab in patients with CRP ≥ 75 mg/L Baricitinib or tofacitinib in patients with CRP ≥ 75 mg/L

## Data Availability

Not applicable as this is a review article not an original research article.
